# Hepatoprotective effect of bone marrow-derived mesenchymal stromal cells in CCl_4_-induced liver cirrhosis

**DOI:** 10.1007/s13205-021-02640-y

**Published:** 2021-01-31

**Authors:** Ashwini P. Aithal, Laxminarayana K. Bairy, Raviraja N. Seetharam, Naveen Kumar

**Affiliations:** 1 Department of Anatomy, Melaka Manipal Medical College (Manipal Campus), Manipal Academy of Higher Education, Manipal, India; 2 Department of Pharmacology, RAK College of Medical Sciences, RAK Medical and Health Sciences University, Ras Al Khaimah, UAE; 3 Stempeutics Research Pvt. Ltd, Manipal, India

**Keywords:** Mesenchymal stromal cells, Multipotent, Liver cirrhosis, Regeneration

## Abstract

Bone marrow mesenchymal stromal cells (BM-MSCs) are multipotent stem cells which are ideal candidates for use in regenerative medicine. The objectives of this study were to evaluate the hepatoprotective effect of BM-MSC and its combination treatment with silymarin in carbon tetrachloride (CCl_4_)-induced liver cirrhosis animal model and to investigate whether tail vein or portal vein infusion was the ideal route for BM-MSC transplantation. 36 female Wistar rats were randomly divided into six groups (*n* = 6): Group 1 (normal control), Group 2 (received only CCl_4_, disease model), Group 3 (CCl_4_ + BM-MSCs through tail vein), Group 4 (CCl_4_ + BM-MSCs through portal vein), Group 5 (CCl_4_ + silymarin), Group 6 (CCl_4_ + BM-MSCs + silymarin). On the 21st day after treatment, blood samples were collected for biochemical estimations. After the experiment, the rats were sacrificed. Liver was dissected out and processed for histopathology and scanning electron microscopy studies. Liver enzyme and marker analysis, histopathological studies indicated that the combination of BM-MSCs and silymarin was effective in treating liver cirrhosis. Transplanted BM-MSCs in combination with silymarin ameliorated the liver tissue damage through their immunoregulatory activities. Among the two routes, the intravenous administration of cells through the tail vein was found to be more effective and safe.

## Introduction

Liver is an important organ of the body which plays an important role in the process of detoxification, immune response, and metabolism, and thus helps in homeostasis. Many factors such as hepatitis, alcoholism, and cholestatic disease can cause hepatic tissue damage leading to hepatic fibrosis, eventually leading to liver cirrhosis and liver failure. Fibrogenesis is a complex process, which involves cellular interplay between hepatocytes, inflammatory cells, biliary epithelial cells, supporting cells like Kuffer cells and stellate cells (Bataller and Brenner 2005); (Lee et al. 2015). During this process, apoptotic hepatocytes induce the activation and increase the proliferation of hepatic stellate cells which then differentiate into myofibroblast. These myofibroblasts then start to produce excessive amounts of extracellular matrix, thereby playing a chief role in the development of fibrosis, leading to liver cirrhosis (Lee et al. 2015).

Therapeutic drugs or treatments which specifically target liver cirrhosis or the process of fibrogenesis are not yet available. Liver transplantation is considered the only available treatment for end-stage liver cirrhosis (van der Helm et al. 2019). But liver transplantation is not a viable option as it involves complications, risks as immune rejection and is dependent on donor availability. Hence, many alternative treatment options including stem cell transplantation are being explored.

Mesenchymal stem cells are multipotent stem cells which can be obtained from many sources such as bone marrow, adipocytes, umbilical cord, placenta, and other embryonic and adult tissues. Bone marrow-derived stromal cells (BM-MSCs) have attracted considerable attention in regenerative medicine owing to their high therapeutic potential. BM-MSCs can be isolated from a small aspirate of bone marrow and cultured without compensation for its potency. Many studies have shown that BM-MSC can differentiate into hepatocyte-like cells. These cells have low inherent immunogenicity and are capable of modulating immunological responses through interaction with various immune cells, thus, improving the safety of using stem cells as therapeutics (Liu et al. 2015a). MSCs improve liver functions by carrying out the functional activities of mature hepatocytes, which are involved in the supportive functions needed for regenerative medicine applications (Zhang and Wang 2013).

This present study aims to evaluate the efficiency and hepatic differentiation potential of BM-MSCs in combination with silymarin in the experimentally induced liver cirrhosis rat model. We further investigated whether tail vein or portal vein infusion was the ideal route for BM-MSC transplantation.

## Materials

Female Wistar albino rats, weighing 140–160 g (4–5 months old), were chosen for this study. Animals were kept in the central animal facility in polypropylene cages, maintained under standard conditions with temperature (26–30 °C) and relative humidity of 40–60%. Rats had continuous access to water and regular rat pellet diet (VRK Laboratory animal feed, Maharashtra, India). Animals were bred for 1 week before the start of the experiment to adapt to the laboratory conditions. Institutional Animal Ethics Committee clearance was obtained prior to the start of the experiment (IAEC/KMC/20/2014) and all experiments were conducted in accordance with the ethical rules of the Ministry of Social Justices and Empowerment, Government of India, and according to the guidelines of Committee for the Purpose of Control and Supervision on Experiments on Animals (CPCSEA).

The stem cells required for the study was obtained from Stempeutics Research Pvt Ltd, Shirdi Sai Baba Cancer Hospital, Manipal. Freshly thawed BM-MSCs were used for intravenous administration. Carbon tetrachloride (CCl_4_) and other chemicals were obtained from Merck Chemicals, Mumbai (India). Silymarin was bought from Sigma Chemical Inc. (USA). The reagents were of analytical grade, stored in a refrigerator, and were equilibrated at room temperature before the start of the analysis.

## Methodology

### Induction of liver cirrhosis in Wistar rats

Carbon tetrachloride (CCl_4_) intoxication was used for the induction of liver cirrhosis in Wistar rats. CCl_4_ was injected i.p. to rats 3 days a week for 28 days at the dose of 1 ml/kg body weight. This will be the same pathological injury that will be noticed in liver cirrhosis.

### Experimental design

Thirty-six female Wistar rats were divided into six groups with six animals in each group. 1st group animals served as normal control and were given distilled water. 2nd group was the disease model, which were administered 1 ml/kg body weight of CCl_4_. 3rd, 4th, and 5th groups were the treatment groups. After the disease confirmation following CCl_4_ intoxication for 28 days, 3rd group animals were treated with silymarin orally (100 mg/ kg body wt.), 4th group animals were treated with BM-MSC intravenously through the tail vein (5.8 million cells in 0.5 ml), 5th group animals were treated with BM-MSC intravenously through the portal vein (5.8 million cells in 0.5 ml), and 6th group animals were treated with a combination of silymarin (100 mg/ kg body wt.) and BM-MSC (5.8 million cells in 0.5 ml through the tail vein). The BM-MSCs were formulated in 0.5 ml PlasmaLyte A (sterile, isotonic solution) injected slowly through the veins. The entire study period was 49 days. At the end of the experimental period, animals were sacrificed by administering an overdose of ketamine, the abdomen region was dissected, and the liver from each rat was rapidly excised and washed thoroughly with isotonic saline. Liver tissue was then processed for histopathological and SEM studies.

### Biochemical parameters

On the 21st day after the treatment, the animals were anesthetized with ketamine following a 12-h fast. Blood was collected in small centrifuge tubes through retro-orbital puncture. Blood obtained was later centrifuged for 10 min at 3000 rpm to separate the serum which was used immediately for liver enzyme and liver function marker analysis. LDH (lactate dehydrogenase), ALP (alkaline phosphatase), ALT (alanine transaminase), AST (aspartate transaminase), albumin, and direct bilirubin were estimated using commercially available kits.

### Histopathology study

The samples of liver tissue from the rats of each group were washed in saline, fixed in 10% formalin solution, dehydrated in ascending grades of alcohol, and embedded in paraffin. Twenty-four hours after block preparation, paraffin sections were obtained on clean glass slides and stained with hematoxylin and eosin stain and Masson’s Trichrome stain. The slides were observed for histopathological changes under a light microscope. Relevant photomicrographs were taken.

### Scanning electron microscopy study

Liver tissue samples (2–3 mm thick) were excised and washed in 0.5 M potassium phosphate buffer at pH 7.2 and 4 °C. Samples were fixed in 2.5% glutaraldehyde solution at 4 °C for 6–8 h, washed with 0.1 M phosphate-buffered saline (PBS) for 15 min. Series of ascending grades of ethyl alcohol were used for 15 min each to dehydrate the samples. Liver samples were then air-dried, coded, and mounted on aluminum stubs. Sections were viewed at 500X magnification on Zeiss Evo 18 scanning electron microscope (Carl Zeiss Microscopy, LLC, Thornwood, NY, USA). Photographs were taken from randomly selected fields.

### Statistical analysis

The data were tabulated and analyzed using SPSS (statistical package for the social science software) version 16.0. Quantitative data were expressed as mean ± SD. Mean values of normal control and treatment groups were compared using one way ANOVA, followed by post hoc Tukey test. A probability value of < 0.05 was considered statistically significant (*p* < 0.05).

## Results

### Effect of BM-MSCs on liver enzyme/liver function marker levels

**Fig. 1:**
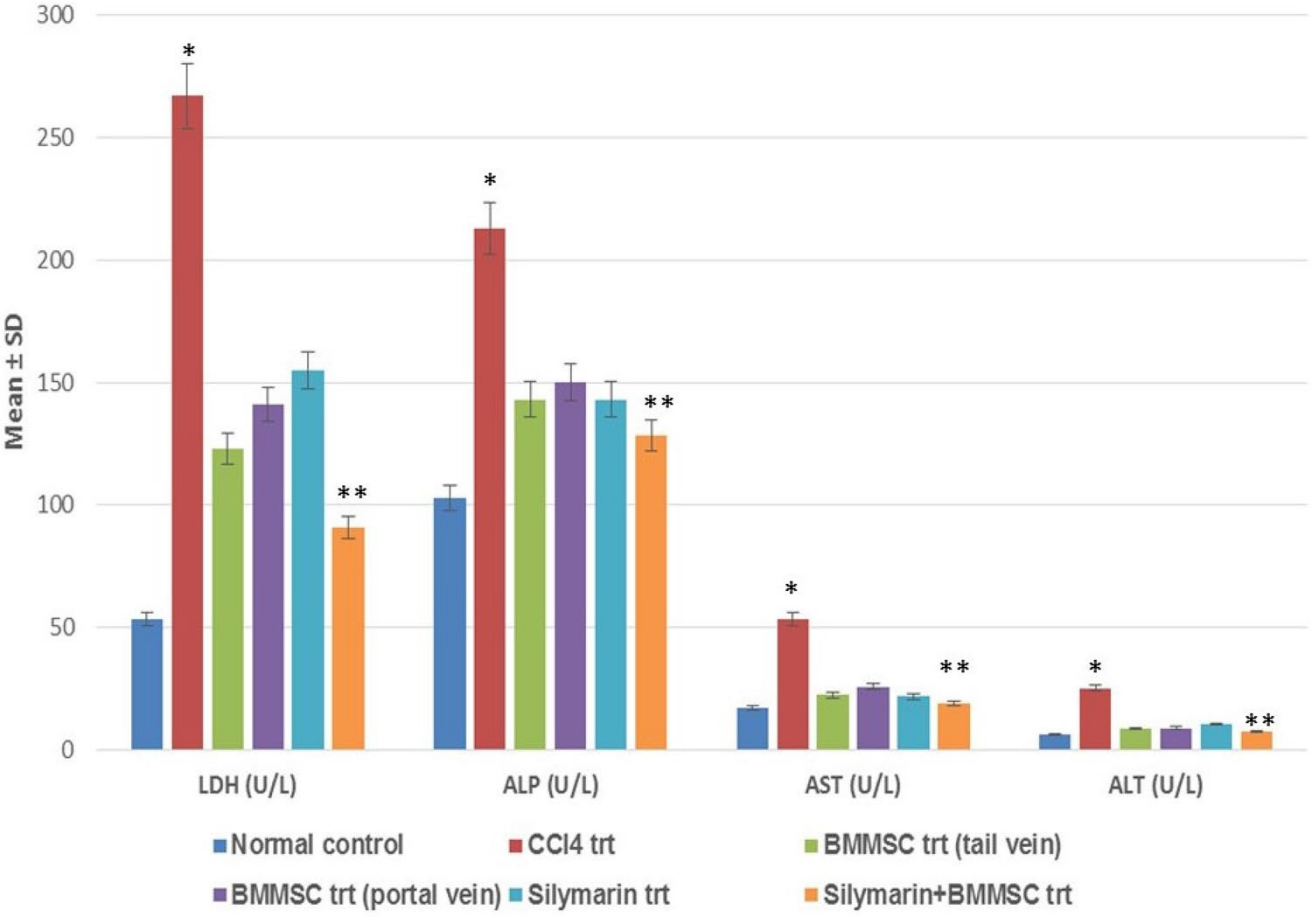
Graph showing the significant difference in liver enzyme levels between the groups. Enzyme levels significantly increased in the CCl_4_-trt group when compared with normal control *(*p* < 0.05); BM-MSC + silymarin trt significantly decreased the enzyme levels which was comparable to normal control, **BM-MSC + silymarin trt vs. CCl_4_ trt (*p* < 0.01); *LDH* lactate dehydrogenase, *AST* aspartate transaminase, *ALP* alkaline phosphatase, *ALT* alanine transaminase

**Fig. 2:**
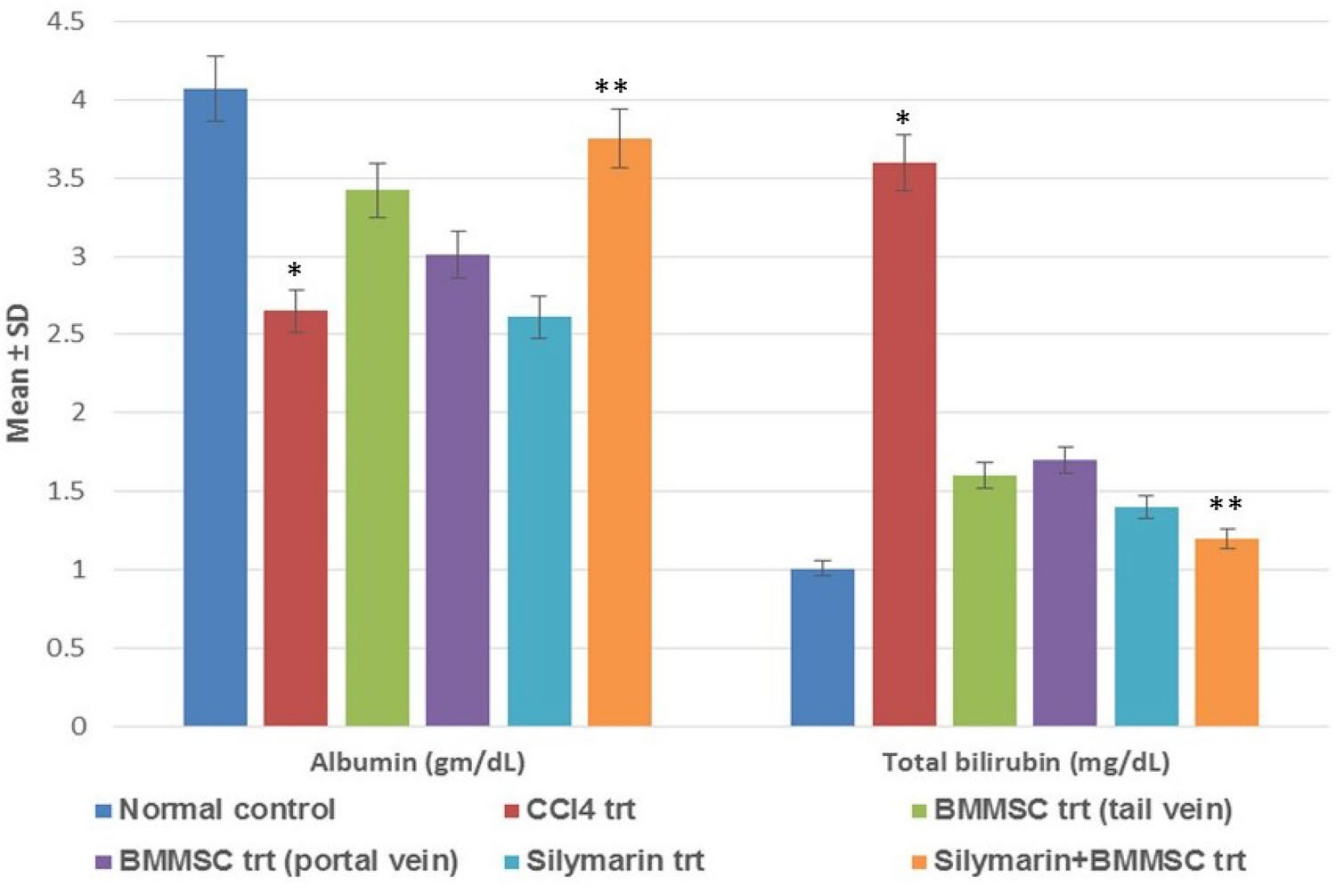
Graph showing the significant difference in liver function marker levels between the groups. Levels of albumin and total bilirubin altered significantly in CCl_4_ trt group when compared with normal control *(*p* < 0.05); BM-MSC + silymarin trt was most effective as it significantly brought the liver function marker levels comparable to normal control values, **BM-MSC + silymarin vs. CCl_4_ trt (*p* < 0.01)

In CCl_4_ disease model group, serum levels of LDH, ALT, AST, ALP, and direct bilirubin were increased significantly, while the albumin level in the serum decreased significantly when compared with the normal control group (*p* < 0.05). This indicates the liver injury caused by CCl_4_ intoxication. It was observed that the treatment with BM-MSCs through the tail vein and portal vein significantly altered the enzyme and marker levels when compared with the CCl_4_-treated group. Among the two routes, results of the tail-vein-transplanted group were better when compared with the infusion through the portal vein. These results indicated the equivalent therapeutic effects of the two routes on liver injury. But, infusion through portal vein could cause venous pressure and embolism, and portal vein transplantation is a difficult/invasive procedure to perform. Therefore, intravenous administration through the tail vein was preferred for the combination treatment (silymarin + BM-MSC) because of its safety and convenience. Results indicated that silymarin + BM-MSC combination treatment was most effective among the treatments as it significantly altered the liver enzyme/marker levels which were comparable to the normal control group (*p* < 0.01) (Figs. 1, 2). The results thus showed that BM-MSC transplantation was safe and facilitated liver function recovery.

### Histopathological analysis

**Fig. 3:**
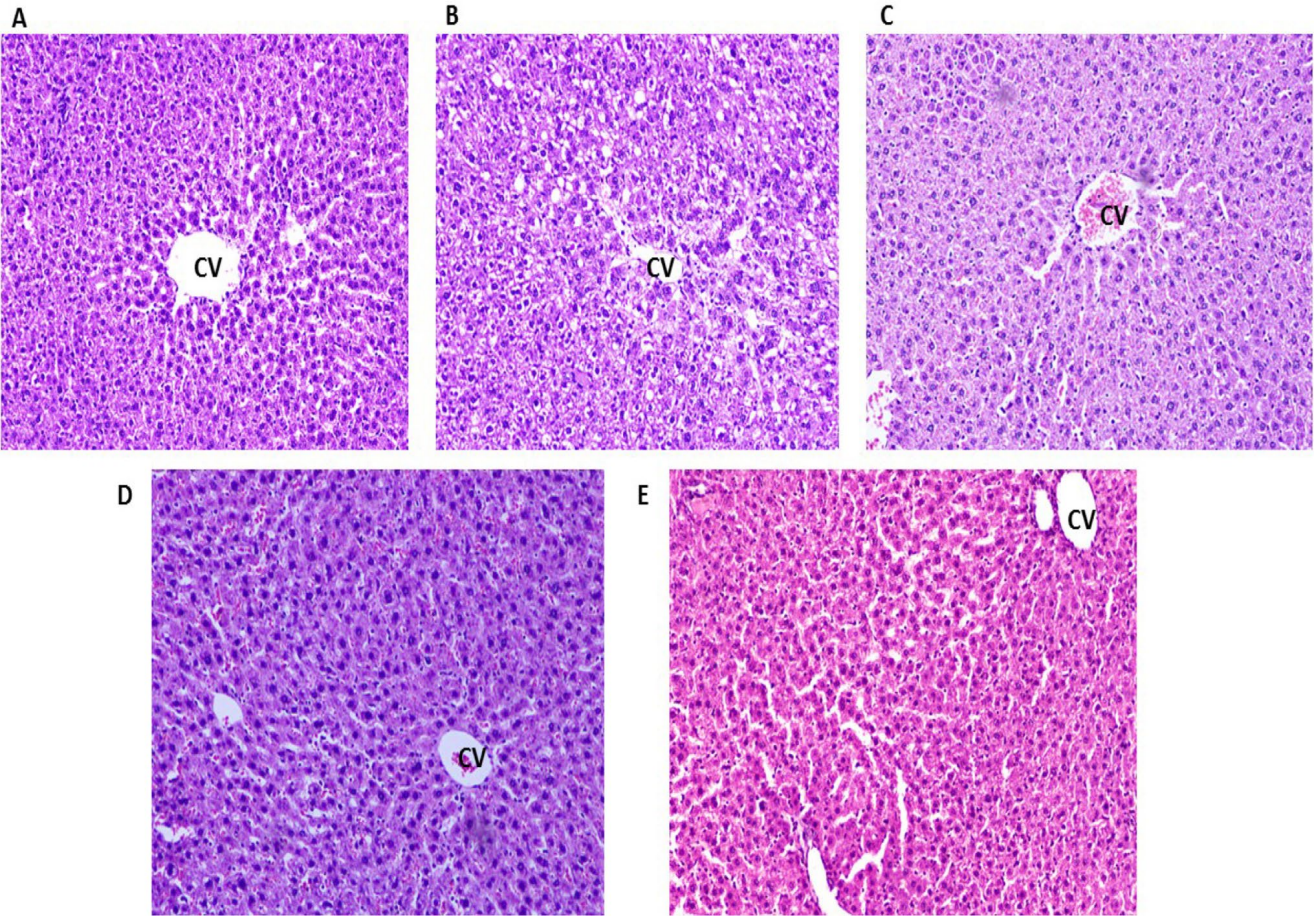
Representative photomicrographs of hematoxylin and eosin-stained liver sections (10 ×). **a** Normal control: showing distinct hepatic cords and narrow sinusoidal spaces; **b** CCl_4_ trt (disease model): showing empty extracellular spaces, disarrangement of hepatic parenchyma with necrosis; **c** BM-MSCs trt and **d** silymarin trt: decreased the sinusoidal spaces, disarrangement of hepatocytes was still evident; **e** BM-MSCs + silymarin trt: showed normal hepatic architecture which was comparable to normal control group. *CV* central vein

CCl_4_-intoxicated group displayed severe fibrosis with collagen fiber infiltration, ballooning of hepatocytes with numerous vacuoles in the liver tissue. Necrosis was observed around the central vein with disarrangement of hepatic sinusoids which indicated liver injury. Silymarin and the BM-MSC treatment showed protection as there was the presence of little collagen fibers and less vacuole formation. Apparently, normal liver architecture was seen in the combination-treated group (Fig. 3) which proved that this combination treatment was more beneficial when compared with the individual treatments.

**Fig. 4:**
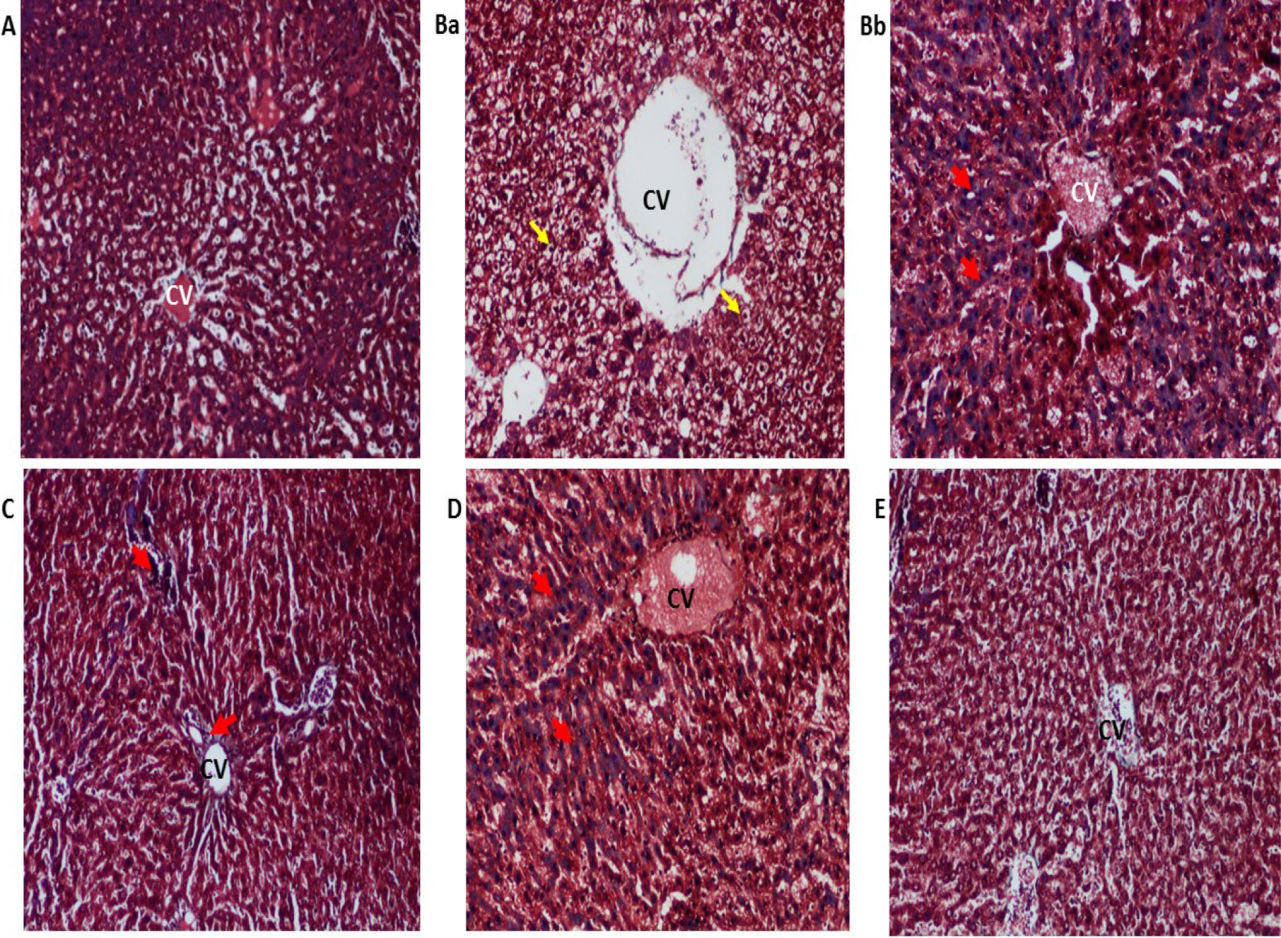
Representative photomicrographs of Masson Trichrome-stained liver sections (10 ×). **a** Normal control: showing normal hepatic parenchyma; **Ba** CCl_4_ trt (disease model) showing vacuole formation, disarrangement of central vein (CV) and hepatocytes with enlarged nucleus (yellow arrow); **Bb** CCl_4_ trt group showing prominent collagen fiber deposition between the hepatocytes (red arrow) (C) BM-MSCs trt and **d** silymarin trt; both sections showed the presence of collagen fibers (red arrow) **e** BM-MSCs + silymarin trt: appearance of normal hepatocytes with narrow sinusoidal spaces around the central vein (CV), with negligible collagen fibers

Masson’s Trichrome is the choice of special stain for the demonstration of collagen fibers. Aniline blue or light green is used to stain the collagen tissue selectively. Both these dyes have higher molecular weight than the other dye Ponceau fuchsin, which is used in this method for the demonstration of cytoplasm and muscle tissues. The dye with the higher molecular weight has a tendency to stain the more easily penetrable collagens. The mordant used in this stain is phosphomolybdic acid which can differentiate the collagens selectively to facilitate them to stain with the specific dye. In this study, aniline blue has been used to stain the collagen; hence, collagen fiber appears blue in color and the cytoplasm as red. The nuclei of the hepatocytes appear blue or black (Jones et al. 2008). MT not only stains type 1 collagen which is normally present in the portal tracts and walls of blood vessels but also highlights the presence and distribution of reactive fibrosis as a result of liver injury. It is used for staging chronic liver diseases and helps to outline the patterns of injury (Krishna 2013). In CCl_4_-treated rats, apoptotic cells were observed with persistent inflammation leading to degenerative swelling of liver cells with a lot of vacuole formation (Fig. 4Ba). Collagen fibers were found distributed extensively all around in the liver tissues especially around the central vein and portal triad areas (Fig. 4Bb). On the other hand, the inflammatory state improved in the stem cell and silymarin-treated groups (Fig. 4c, d). However, the presence of collagen fibers was still seen in the liver tissue. Normal hepatic architecture with very few collagen fibers, no fibrosis bridge, and normal arrangement/appearance of hepatocytes was observed in the combination-treated group which was comparable to the liver section of normal control rats (Fig. 4e).

### Scanning electron microscopy analysis

**Fig. 5:**
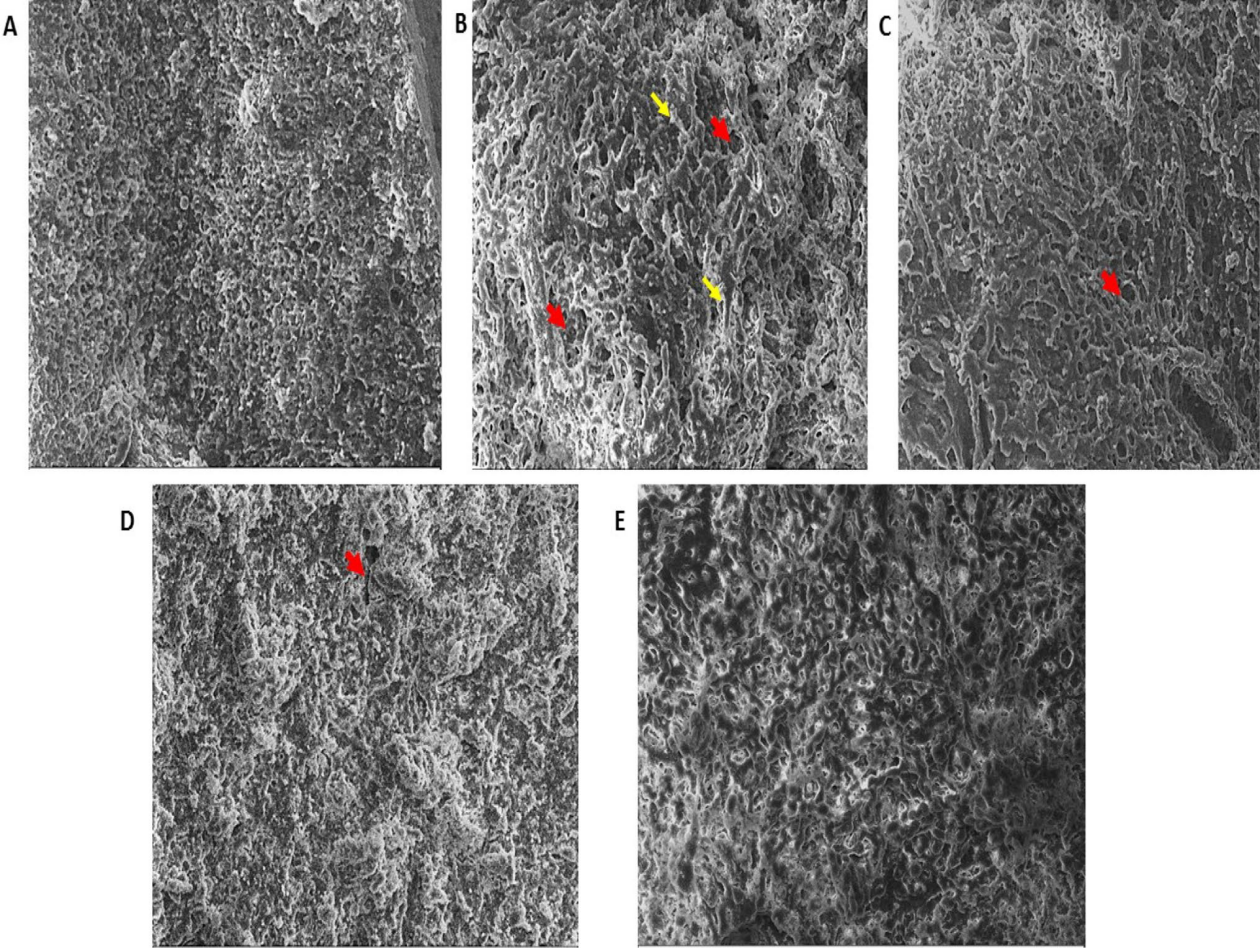
Scanning electron micrographs of rat liver (500 ×). **a** Normal control: showing smooth surface of liver with distinct hepatocytes and very few collagen fibers; **b** CCl_4_ trt: most of the hepatocytes appear disarranged with formation of pseudo-lobules (red arrow) formed due to the presence of thick collagen fiber bands (yellow arrow); **c** BM-MSCs trt and **d** silymarin trt: both showed the presence of collagen fibers; **e** silymarin + high dose BM-MSCs trt group: showing well-defined liver parenchyma with reduction in collagen fibers and normal liver parenchyma

Surface morphology of the CCl_4_-treated group showed irregular surface of the liver tissue section with necrosis of hepatocytes and dilated blood sinusoids. There was formation of cyst-like structures known as the pseudo-lobules which were formed by a network of fibrous tissue. Bundles of collagen fibers were seen in the extracellular spaces of the liver tissue. Electron micrographs of BM-MSCs and silymarin treatment showed some hepatoprotective activity; however, few hepatocytes still appeared degenerated with irregular surface and presence of collagen fiber bands. Combination treatment of silymarin and BM-MSCs markedly reduced the extracellular spaces and collagen fiber bands. The surface of the liver tissue was smooth; hepatocytes appeared normal with narrow sinusoids and absence of vacuoles (Fig. 5).

## Discussion

The therapeutic properties of BM-MSCs in liver cirrhosis reflected in the present study can be related to their hepatocyte-like differentiation capacity, their antifibrotic, immune-modulatory, and antioxidant activities (Ewida et al. 2017). Mesenchymal stem cells express high potential therapeutic value due to their self-renewal ability and multipotent differentiation, and low immunogenicity (Jang et al. 2015). On the other hand, the therapeutic efficacy of BM-MSCs in managing liver cirrhosis and the mechanisms involved in the process are still controversial. Many studies have proved that BM-MSCs can ameliorate hepatic fibrosis while others have proposed that BM-MSCs can aggravate liver fibrosis (Liu et al. 2015b). The above-said controversies might be attributed to the varied delivery routes by which BM-MSCs have been transplanted into the host tissue. The intravenous route through the tail vein is considered to be the most safest and effective way of stem cell transplantation, which has already been proved by us in a previous study (Aithal et al. 2017a). In the current study, we tried two different routes of stem cell transplantation and found that the intravenous route through the tail vein is least invasive and showed better hepatic protection when compared with the portal vein route. A similar finding was observed by Zhao et al., who demonstrated that intravenous injection was the most effective among intravenous, intrahepatic, and intraperitoneal injection routes (Zhao et al. 2012). Thus, careful administration of BM-MSCs through the intravenous route through the tail vein might be one of the prerequisites and effective strategies for the treatment of liver cirrhosis.

In our experimental study, we used an animal model of liver cirrhosis using CCl_4_ as it is one of the most extensively used toxins for the induction of liver cirrhosis which resembles the same pathology of liver damage in humans (Abdel Aal et al. 2019). Significant changes were noticed in liver enzyme levels, i.e., LDH, ALT, AST, ALP, and liver function marker (albumin, bilirubin, total protein) levels in BM-MSC and combination treatment groups as compared with that in the CCl_4_-treated group. This finding is in agreement with earlier studies which report that transplanted MSCs could restore the serum liver function marker and liver enzyme levels significantly thereby reducing the liver fibrosis in the injured rat liver (Aithal et al. 2017b; Nasir et al. 2013). Silybum marianum and its ingredient silymarin exert hepatoprotective effects against liver diseases; CCl_4_-induced liver damage and is used as a standard drug control (Mohammed et al. 2016); (Abbasi et al. 2016). It was observed in previous studies done by us that the combination treatment of BM-MSC and silymarin proved to be more beneficial when compared with the single treatments (Aithal et al. 2017b). Studies have also shown that the regeneration of hepatocytes following MSC treatment could be observed as early as on day 8 (van der Helm et al. 2019). Thus, in the present study, we have changed the dose of BM-MSCs and reduced the duration of the experiment to 21 days. This also proved to be equally efficacious which was evident from the significant hepatoprotective activity of the combination treatment. These findings suggest that the combination treatment acted synergistically and augmented the hepatic environment, leading to increased MSC survival in the host tissue, thereby leading to better differentiation of MSCs into hepatocytes and significantly improving the hepatic function which was demonstrated by restored serum liver enzyme levels which were comparable to the normal control animals.

Fibrosis is triggered by a chronic injury to the liver which is caused by a series of events that include damage to the endothelial barrier; release of a major fibrogenic cytokine TGF-b1; production of inflammatory cells and activation of collagen-producing cells; induction of reactive oxygen species; matrix activation of myofibroblasts; finally, in the absence of continuous injury, there is a reversal of fibrosis (Kisseleva and Brenner 2008). When there is no reversal, the fibrosis leads to the development of liver cirrhosis. BM-MSCs may attenuate acute liver inflammation and consequent hepatocyte damage by secreting various soluble factors and trophic factors such as growth factors, few cytokines, and chemokines which play key therapeutic roles in regenerative medicine (Eom et al. 2015). MSCs modulate the production of inflammatory cytokines and other inflammation-related molecules which not only reduce the inflammation, apoptosis of cells, and fibrosis of damaged tissues but also stimulates angiogenesis and tissue cell regeneration, thereby creating a hepato-protective environment in the liver (Gazdic et al. 2017).

Histopathological examination of liver sections in CCl_4_-treated group showed different alterations in the hepatic lobular architecture in the form of detached endothelial lining of the central vein, congested blood sinusoids, and portal vein. Most of the hepatocytes showed vacuolated cytoplasm. It has been shown in previous studies that prolonged administration of CCl_4_ leads to the occurrence of centrizonal necrosis causing liver fibrosis, cirrhosis, and hepatocellular carcinoma (Mohammed et al. 2016). The observed toxic effects of CCl_4_ intoxication are related to the biotransformation of CCl_4_ into a more destructive trichloromethyl peroxy radical (CCl_3_OO) in the presence of oxygen. Masson Trichrome-stained sections of CCl_4_ group showed a marked increase in collagen deposition around the central vein and portal area. Liver cirrhosis results from apoptosis of the hepatocytes in conjunction with excessive deposition of collagen fibers and other components of the extracellular matrix, which is a characteristic of most types of chronic liver diseases (Abdel Aal et al. 2019). Combination treatment of BMMSCs and silymarin markedly reduced the deposition of collagen fibers and vacuoles thereby effectively reducing the histopathological changes.

BM-MSCs also increase the expression of matrix metalloproteinases (MMPs) and decrease the tissue inhibitors of MMPs (TIMPs) and these alterations are largely linked to fibrosis resolution. Studies have shown that transplanted BM-MSCs can degrade collagen fibers significantly with strong expression of MMPs, especially MMP-9 (Sakaida et al. 2004). Higashiyama et al. have reported that MMP-9 expressed by BM-MSCs release soluble Kit-ligand, which might be related to the transfer of the stem cells to the proliferative niche (Higashiyama et al. 2007). Therefore, MMP-9 could play an important role in the degradation of extracellular matrix and also by releasing some factors, e.g., soluble Kit-ligand, leading to enhanced differentiation and proliferation capacity of transplanted BM-MSCs in liver inflammation induced by CCl_4_ (Sakaida et al. 2004). MSCs act by eliminating the deposition of extracellular matrix directly or by an indirect way by differentiation or fusion with the hepatocytes, or immunological regulation, inducing paracrine effects, thereby contributing to the degradation of scar tissue and promotion of myofibroblast apoptosis. All the above-said potential mechanisms are involved in the therapeutic effects of BM-MSC in liver cirrhosis models (Hu et al. 2019).

The efficacy of transplanted BM-MSCs within the host liver tissue still remains a serious concern. Transplanted BM-MSCs encounter toxic, inflammatory microenvironment in the host tissue which causes many MSCs to undergo cell death; thus, only a small number of MSCs have the potential to migrate into specific injury sites. It is found that the microenvironment around MSCs plays a crucial role in the activities of MSC as they promote the proliferation and differentiation of MSCs in the host tissue (Hu et al. 2019). Thus, augmenting the BM-MSC capability for liver repair by enhancing the liver tissue environment can lead to increased MSC engraftment. A previous study has proved the beneficial effect of the combination of MSCs and IL-6 treatment which enhanced the survival of hepatocytes, thereby leading to the increased efficacy of MSC therapy (Aithal et al. 2019). In the current study also this protective effect was found. Silymarin can exert an antioxidant effect on the hepatocytes. Therefore, it can be assumed that improvement in the hepatic microenvironment by virtue of increased hepatocyte protection by silymarin would enable the increased BM-MSC homing, enhanced survival of these cells, and facilitate better hepatic repair which was evident in liver sections which showed reduced fibrosis and cell apoptosis. The development of novel cell therapy strategies for liver cirrhosis is in constant evolution, which is driven by the developing nature of the various biotechnology processes used to unveil the molecular aspects and cell interactions within the hepatic niche in health and disease (Pinheiro et al. 2019). Many concerns still remain regarding the low transplantation and fibrogenic potential of BM-MSC along with oncogenic risk (Alfaifi et al. 2018). Therefore, many preclinical studies have to be performed to unravel the underlying mechanism of MSC therapy before their large-scale application in clinical settings. The limitations of the study were that the underlying working mechanisms involved in the regeneration process were not assessed as the present study was an observational study to evaluate the therapeutic effects of BM-MSC on the reversal of fibrogenesis.

## Conclusion

In conclusion, we have observed that transplanted BM-MSCs ameliorated liver tissue damage through their immunoregulatory activities. There have been extensive efforts and diverse approaches to enhance the efficacy and homing efficiency of transplanted BM-MSCs. Thus, the novel strategy used in the present study to combine the BM-MSC with silymarin might be a promising tool to treat liver cirrhosis and in regenerative medicine.

## References

[CR1] Abbasi BH, Ali H, Yücesan B (2016). Evaluation of biochemical markers during somatic embryogenesis in Silybum marianum L. 3 Biotech.

[CR2] Abdel Aal S, Abdelrahman S, Raafat N (2019). Comparative therapeutic effects of mesenchymal stem cells versus their conditioned media in alleviation of CCL4-induced liver fibrosis in rats: histological and biochemical study. J Med Histol.

[CR3] Aithal AP, Bairy LK, Seetharam RNKN (2017). Therapeutic potential of human bone marrow-derived mesenchymal stromal cells in combination with silymarin against carbon tetrachloride induced liver cirrhosis in Wistar rats. JKIMSU.

[CR4] Aithal AP, Bairy LK, Seetharam RN (2017). Safety assessment of human bone marrow-derived mesenchymal stromal cells transplantation in Wistar rats. J Clin Diagnostic Res.

[CR5] Aithal AP, Bairy LK, Seetharam RN, Rao MKG (2019). Human bone marrow-derived mesenchymal stromal cells in combination with silymarin regulate hepatocyte growth factor expression and genotoxicity in carbon tetrachloride induced hepatotoxicity in Wistar rats. J Cell Biochem.

[CR6] Alfaifi M, Eom YW, Newsome PN, Baik SK (2018). Mesenchymal stromal cell therapy for liver diseases. J Hepatol.

[CR7] Bataller R, Brenner DA (2005). Liver fibrosis. J Clin Invest.

[CR8] Eom YW, Shim KY, Baik SK (2015). Mesenchymal stem cell therapy for liver fibrosis. Korean J Intern Med.

[CR9] Ewida SF, Abdou AG, El-Rasol Elhosary AA, El-Ghane Metawe SA (2017). Hepatocyte-like versus mesenchymal stem cells in CCl 4-induced liver fibrosis. Appl Immunohistochem Mol Morphol.

[CR10] Gazdic M, Arsenijevic A, Markovic BS (2017). Mesenchymal stem cell-dependent modulation of liver diseases. Int J Biol Sci.

[CR11] Higashiyama R, Inagaki Y, Hong YY (2007). Bone marrow-derived cells express matrix metalloproteinases and contribute to regression of liver fibrosis in mice. Hepatology.

[CR12] Hu C, Zhao L, Duan J, Li L (2019). Strategies to improve the efficiency of mesenchymal stem cell transplantation for reversal of liver fibrosis. J Cell Mol Med.

[CR13] Jang YO, Jun BG, Baik SK (2015). Inhibition of hepatic stellate cells by bone marrow-derived mesenchymal stem cells in hepatic fibrosis. Clin Mol Hepatol.

[CR14] Jones ML, Bancroft JD, Gamble M (2008) Connective tissues and stains. In: Theory and practice of histological techniques, 6th edn. Churchill Livingstone, London

[CR15] Kisseleva T, Brenner DA (2008). Mechanisms of fibrogenesis. Exp Biol Med.

[CR16] Krishna M (2013). Role of special stains in diagnostic liver pathology. Clin Liver Dis.

[CR17] Lee YA, Wallace MC, Friedman SL (2015). Pathobiology of liver fibrosis: a translational success story. Gut.

[CR18] Liu Y, Yang X, Jing Y (2015). Contribution and Mobilization of mesenchymal stem cells in a mouse model of carbon tetrachloride-induced liver fibrosis. Sci Rep.

[CR19] Liu W, Song F, Ren L (2015). The multiple functional roles of mesenchymal stem cells in participating in treating liver diseases. J Cell Mol Med.

[CR20] Mohammed A, Abd Al Haleem EN, El-Bakly WM, El-Demerdash E (2016). Deferoxamine alleviates liver fibrosis induced by CCl4 in rats. Clin Exp Pharmacol Physiol.

[CR21] Nasir GA, Mohsin S, Khan M (2013). Mesenchymal stem cells and Interleukin-6 attenuate liver fibrosis in mice. J Transl Med.

[CR22] Pinheiro D, Dias I, Ribeiro Silva K (2019). Mechanisms underlying cell therapy in liver fibrosis: an overview. Cells.

[CR23] Sakaida I, Terai S, Yamamoto N (2004). Transplantation of bone marrow cells reduces CCl4-induced liver fibrosis in mice. Hepatology.

[CR24] van der Helm D, Barnhoorn MC, de Jonge-Muller ESM (2019). Local but not systemic administration of mesenchymal stromal cells ameliorates fibrogenesis in regenerating livers. J Cell Mol Med.

[CR25] Zhang Z, Wang FS (2013). Stem cell therapies for liver failure and cirrhosis. J Hepatol.

[CR26] Zhao W, Li JJ, Cao DY (2012). Intravenous injection of mesenchymal stem cells is effective in treating liver fibrosis. World J Gastroenterol.

